# Whole-Genome Sequence of Duck Tembusu Virus Strain DTMUV/CH/2014, Isolated in China

**DOI:** 10.1128/genomeA.01657-15

**Published:** 2016-01-28

**Authors:** Xinrong Zhou, Tiansheng Zhang, Deping Song, Tao Huang, Qi Peng, Yanjun Chen, Anqi Li, Fanfan Zhang, Qi Wu, Yu Ye, Yuxin Tang

**Affiliations:** Department of Preventive Veterinary Medicine, College of Animal Science and Technology, Jiangxi Agricultural University, Nanchang, Jiangxi, China

## Abstract

The whole-complete genome sequence of a strain of duck tembusu virus (DTMUV), DTMUV/CH/2014, affecting layer ducks in China, was determined and characterized. Compared with DTMUV sequences available in GenBank, DTMUV/CH/2014 has 3 amino acid mutations located in the capsid, prM, and NS3 genes of DTMUV/CH/2014.

## GENOME ANNOUNCEMENT

In April 2010, a novel disease characterized by acute severely decreased egg production and neurological signs mainly affecting ducks and geese emerged on a duck farm in China. Subsequently, the causative agent was identified as tembusu virus (TMUV), a member of family *flavivirus* (1–3). Since then, cases of TMUV infections of ducks have frequently been reported in many duck-raising provinces in China (3–7). TMUV can infect all species of duck with almost 100% morbidity and up to 12% mortality (1, 2, 8, 9).

In 2014, laying ducks on a duck farm in southern China showed high fever, decreased egg production, and neurological signs. Samples were collected and virus isolation was performed using a conventional procedure (10). According to the epidemiology, clinical signs, and gross lesions of the affected ducks, the causative agent was initially diagnosed as TMUV, and was subsequently identified by conventional reverse transcription-PCR (RT-PCR) and sequencing established in our laboratory. To determine the complete genome of one representative TMUV isolate associated with the disease, ten pairs of overlapping PCR primers based on the published complete genome sequences were designed to amplify the complete genome of the isolated strain. Then, ten overlapping fragments amplified by RT-PCR using the M-MLV reverse transcriptase and *Ex Taq* polymerase (TaKaRa, Dalian, China) with the designed primers were obtained. The 5′- and 3′-terminal sequences were amplified using the SMARTer RACE cDNA amplification kit (Clontech, Beijing, China). Afterwards, the PCR products were purified, cloned into the pMD-18T vectors (TaKaRa, Dalian, China), and then sent to Sangon, a commercial sequencing company in Shanghai, China for sequencing. Using the SeqMan software on DNAStar Lasergene v7.10 (DNAStar, Inc., Madison, WI), the complete genome sequence of this strain, designated DTMUV/CH/2014, was assembled and annotated.

The full-length genome sequence of DTMUV/CH/2014 was 10,991 nucleotides (nt) in length excluding the poly(A) tail and contained a long open reading frame (ORF) (from 96 nt to 10,373 nt) encoding a polyprotein of 3,425 amino acids (aa), including three structural proteins (capsid, prM, and envelope proteins), and seven nonstructural proteins (NS1, NS2A, NS2B, NS3, NS4A, NS4B, and NS5) (2). Sequence comparative analysis with available DTMUVs demonstrated that DTMUV/CH/2014 had 97.8% to 99.3% homology at the nucleotide level and 95.5% to 99.2% homology at the amino acid level, respectively. DTMUV/CH/2014 had 3 aa mutation sites K68E, G179A, and D1963N in the capsid, prM, and NS3 genes of DTMUV/CH/2014, respectively, which might be associated with the alteration of antigenicity, tropism, and virulence of the strain. A phylogenetic tree based on the whole-genome sequence revealed that DTMUV/CH/2014 and duck/WR/China/2010 (accession no. JX196334) fall into the same clade.

In conclusion, the complete genome sequence of DTMUV/CH/2014 determined in this study will contribute to further investigation of the epidemiology and evolution of the tembusu virus.

### Nucleotide sequence accession number.

The complete genome sequence of the DTMUV/CH/2014 strain determined was deposited to GenBank under the accession number KP096415.
